# Sensory Arsenal on the Stinger of the Parasitoid Jewel Wasp and Its Possible Role in Identifying Cockroach Brains

**DOI:** 10.1371/journal.pone.0089683

**Published:** 2014-02-26

**Authors:** Ram Gal, Maayan Kaiser, Gal Haspel, Frederic Libersat

**Affiliations:** 1 Department of Life Sciences and the Zlotowski Center for Neuroscience, Ben-Gurion University of the Negev, Beer-Sheva, Israel; 2 Department of Biological Sciences, New Jersey Institute of Technology, Newark, New Jersey, United States of America; Universitaet Regensburg, Germany

## Abstract

The parasitoid jewel wasp uses cockroaches as live food supply for its developing larva. To this end, the adult wasp stings a cockroach and injects venom directly inside its brain, turning the prey into a submissive ‘zombie’. Here, we characterize the sensory arsenal on the wasp’s stinger that enables the wasp to identify the brain target inside the cockroach’s head. An electron microscopy study of the stinger reveals (a) cuticular depressions innervated by a single mechanosensory neuron, which are presumably campaniform sensilla; and (b) dome-shaped structures innervated by a single mechanosensory neuron and 4–5 chemosensory neurons, which are presumably contact-chemoreceptive sensilla. Extracellular electrophysiological recordings from stinger afferents show increased firing rate in response to mechanical stimulation with agarose. This response is direction-selective and depends upon the concentration (density) of the agarose, such that the most robust response is evoked when the stinger is stimulated in the distal-to-proximal direction (concomitant with the penetration during the natural stinging behavior) and penetrating into relatively hard (0.75%–2.5%) agarose pellets. Accordingly, wasps demonstrate a normal stinging behavior when presented with cockroaches in which the brain was replaced with a hard (2.5%) agarose pellet. Conversely, wasps demonstrate a prolonged stinging behavior when the cockroach brain was either removed or replaced by a soft (0.5%) agarose pellet, or when stinger sensory organs were ablated prior to stinging. We conclude that the parasitoid jewel wasp uses at least mechanosensory inputs from its stinger to identify the brain within the head capsule of the cockroach prey.

## Introduction

The parasitoid jewel wasp (*Ampulex compressa*) uses live cockroaches as food supply for its developing larva [1]–[6]. To achieve this, the wasp stings a cockroach twice: first in the thorax and then in the head. The first sting paralyzes the prey’s front legs for 3–5 min, during which the wasp directs its stinger through the cockroach’s neck and into its head. The latter sting induces a long-lasting lethargic state, during which the cockroach demonstrates a dramatically reduced drive to self-initiate movement. This enables the wasp to walk the ‘zombie’ cockroach into a nest, lay an egg on its leg and seal the nest with leaves and pebbles collected nearby. The wasp’s larva later hatches, feeds on the live cockroach and ultimately pupates inside its abdomen.

**Figure 1:**
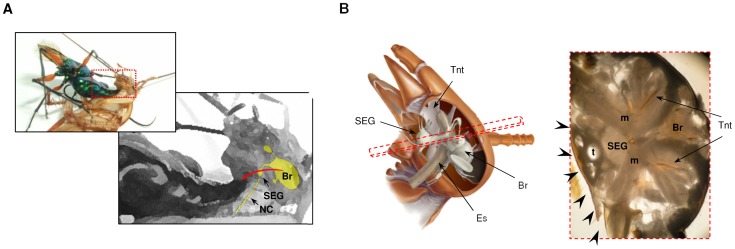
The jewel wasp stings a cockroach into the brain. (**A**) A photograph and a diagram showing the presumable trajectory of the wasp’s stinger (red) inside the head of its cockroach host. The wasp holds the cockroach by the pronotum while bending the abdomen towards the cockroach’s head, inserting the stinger through the soft neck cuticle. The central nervous system of the cockroach is depicted in yellow. Br: Brain, SEG: subesophageal ganglion, NC: neck connectives. (**B**) Left: a lateral view of the cockroach head demonstrating the central nervous system (brain (Br) and SEG), the esophagus (Es) and the internal head skeleton (tentorium; Tnt). Right: light micrograph of a cross section of the head (taken from the plane shown as a dashed rectangle on the left), showing the brain, SEG, internal skeleton, trachea (t) and muscles (m). Different possible points of entry of the stinger through the soft neck cuticle are illustrated by arrowheads.

To induce the lethargic state the wasp must inject venom through its stinger, a modified ovipositor, into the head ganglia of its cockroach prey [7]. This is accomplished by inserting the stinger through the cockroach’s “neck”, i.e. the ventral membranous tissue connecting the head and thorax (Fig. 1A). The exact point of entry of the stinger through this neck cuticle and into the head capsule, however, depends on the posture of the initial encounter and is therefore variable (Fig. 1B, arrowheads). Thus, the stinger must pierce from different locations in the neck and through different head-born tissue (including muscles, trachea, internal skeleton etc.) until it reaches its ultimate target, the supraesophageal ganglion (‘brain’), and then penetrates through the protective ganglionic sheath (Fig. 1B). We have previously shown that this process may involve sensory inputs, as removing the cockroach’s brain prior to a wasp’s sting significantly prolongs the head-sting duration [8], [9]. It is therefore plausible that sensory organs on the stinger, which in other wasps serve to locate, select and evaluate the suitability of the host (e.g., [10]–[22]), have evolved in the jewel wasp to identify the brain inside the head capsule of the host and discriminate it from other tissues. The current study aimed at characterizing such possible sensory organs and the mechanism by which the wasp uses them to recognize the brain during the head-sting.

## Results and Discussion

**Figure 2:**
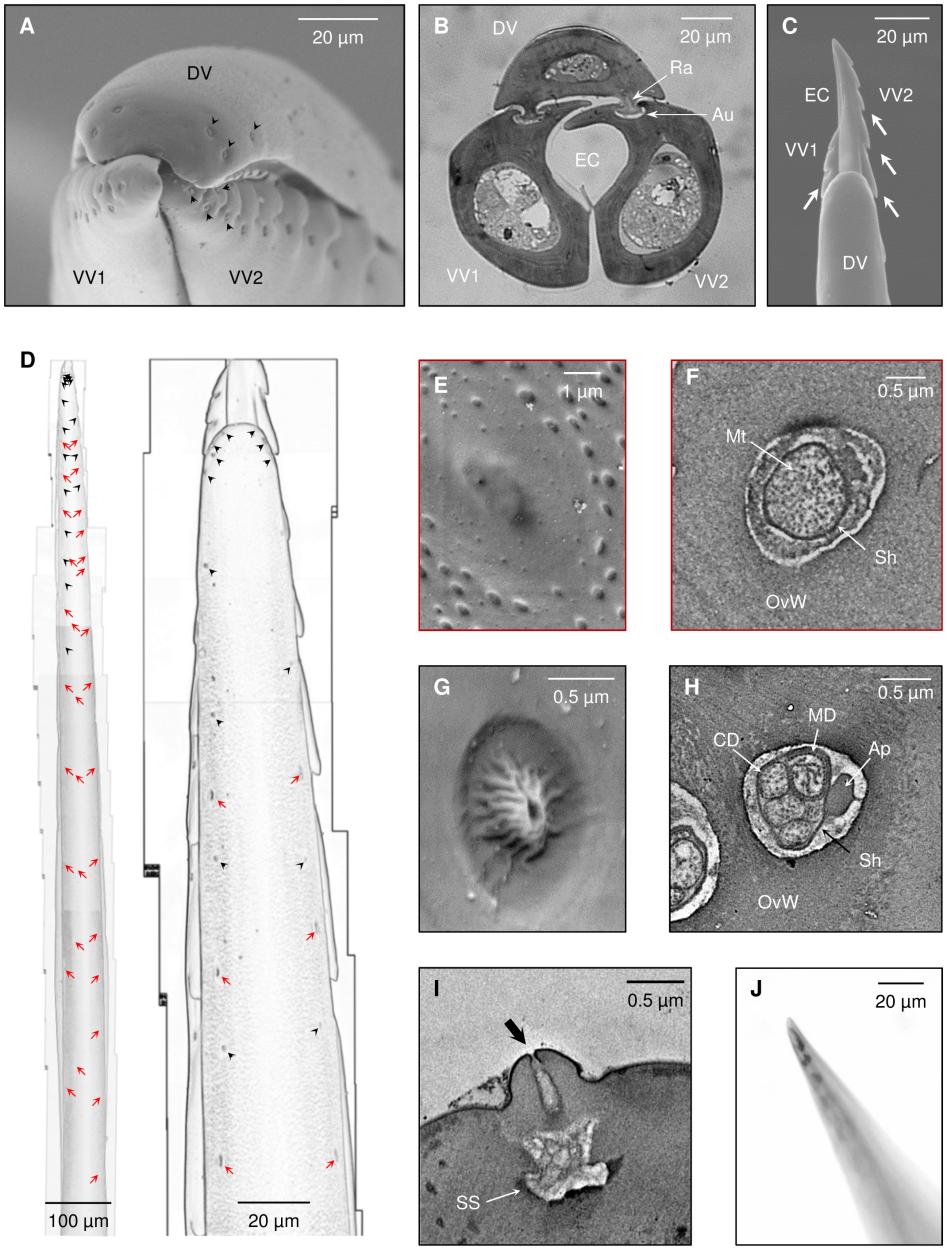
The stinger possesses mechanosensory and dual mechano-chemosensory organs. (**A**) Frontal view of the tip of the stinger (Scanning Electron Micrograph; SEM). *DV*: dorsal valve, *VV1/2*: first/second ventral valve. Dome-shaped sensilla (*arrowheads*) can be seen at the apex of the DV (two opposing triplets) and between serrations of the two VVs. (**B**) Cross-section of the stinger (light micrograph) showing the DV and two VVs enclosing the egg canal (*EC*). The tongue-and-groove structure of the rachis (*Ra*) and aulax (*Au*) enables intricate movements of the valves relative to each other. (**C**) Dorsal view of the stinger (SEM). The VVs in this image are extended distally to reveal their serrations (*arrows*) and a part of the EC. (**D**) Outlines of the stinger (distal part enlarged on the right) showing the distribution of different sensilla along the DV. *Red arrows* indicate the position of campaniform sensilla; *black arrowheads* indicate the position of dome-shaped sensilla. (**E**) External morphology of one campaniform sensillum (SEM). (**F**) A mechanosensory dendrite innervating a campaniform sensillum (Transmission Electron Micrograph; TEM). *OvW*: ovipositor wall, *MT*: microtubules, *Sh*: dendritic sheath. (**G**) External morphology of one dome-shaped sensillum (SEM). (**H**) A bundle of 4 chemosensory dendrites (*CD*) and 1 mechanosensory dendrite (*MD*) innervating a dome-shaped sensillum (TEM). *OvW*: ovipositor wall; *Ap*: apodeme. *Sh*: sheath. (**I**) Longitudinal section (TEM) through one dome-shaped sensillum demonstrating the apical pore (*arrow*) and sensillar sinus (*SS*). (**J**) Silver nitrate staining (light micrograph) of the stinger showing penetration of the tracer (*black staining*) through the pores of dome-shaped sensilla.

The jewel wasp’s stinger (Fig. 2) is approximately 2 mm in length, which is long enough to reach the cockroach’s brain when inserted from the neck [7]. As in many other parasitoids (e.g., [11], [14]), the stinger comprises three appendages (‘valves’) – an unpaired dorsal valve and a pair of ventral valves – which together enclose the egg canal and venom injection apparatus (Fig. 2A–C). A tongue-and-groove arrangement (the rachis and aulax; Fig. 2B) allows movement of the different valves relative to each other and enables intricate steering maneuvers [19]. Between 11 and 13 saw-teeth-like serrations reach 600–700 µm proximally from the apex on each of the ventral valves, whereas the dorsal valve is smooth and devoid of any serrations (Fig. 2A, C). Parasitoid wasps typically use such serrations to anchor the ovipositor inside the host’s integument during stinging, oviposition or transportation [13], [23]. The jewel wasp, however, is an ectoparasitoid and its stinger only penetrates through the cockroach’s integument during the stinging process. Hence, it is reasonable to assume that these serrations anchor the ventral valves inside the cockroach’s head capsule as the distal part of the dorsal valve penetrates through the ganglionic sheath and into the cockroach’s brain. The dorsal valve thus appears to play a more active role in the brain-recognition process.

An Electron Microscopy study of the distal parts of the stinger reveals two morphologically-distinct sensilla-like cuticular structures (Fig. 2D). According to comprehensive descriptions of similar structures on the stinger of other parasitic wasps [22]–[26] and on ultrastructural data (see below), we characterized these structures as campaniform sensilla (CS) and “dome-shaped” (DS) sensilla. Approximately 30–35 CS are distributed along the distal half of the dorsal valve, whereas distal parts of the ventral valves are devoid of CS. On the dorsal valve, CS are typically arranged as bilateral pairs (more distally) or triplets (more proximally) (Fig. 2D, red arrows) and appear as shallow depressions within the cuticle, often with a small molting pore on the surface of the sensillum (Fig. 2E). Each CS is innervated by a single mechanosensory dendrite with numerous microtubules (Fig. 2F), indicative of a mechanoreceptive function [24], [25].

DS sensilla are distributed along both the dorsal and ventral valves, with density increasing towards the apex (Fig. 2D, black arrowheads). On the distal part of the dorsal valve DS sensilla are characteristically distributed between CS along the longitudinal axis of the valve, or in two opposing triplets on the stinger’s apex (Fig. 2A, D; and see also [11]). On the ventral valves, DS sensilla are characteristically distributed with one sensillum occurring between each two serrations, and an extra sensillum between the first and second serrations (Fig. 2A). They have a distinct external morphology, as each DS sensillum appears as a dome situated within an oval groove (Fig. 2G), and each dome possesses a wide apical pore (Fig. 2G, I). Concomitant with their presumed contact-chemosensory nature (as was suggested for other parasitoid wasps, e.g. [24]–[26]), DS sensilla are innervated by one mechanoreceptive neuron and 4–5 chemoreceptive neurons (Fig. 2H). The mechanoreceptive neuron is associated with an apodeme (Fig. 2H), suggesting a stretch-receptor function [25]. The apical pore allows the penetration of silver nitrate into the sensillar sinus (Fig. 2J), suggesting a chemosensory function in addition to mechanosensation [26].

**Figure 3:**
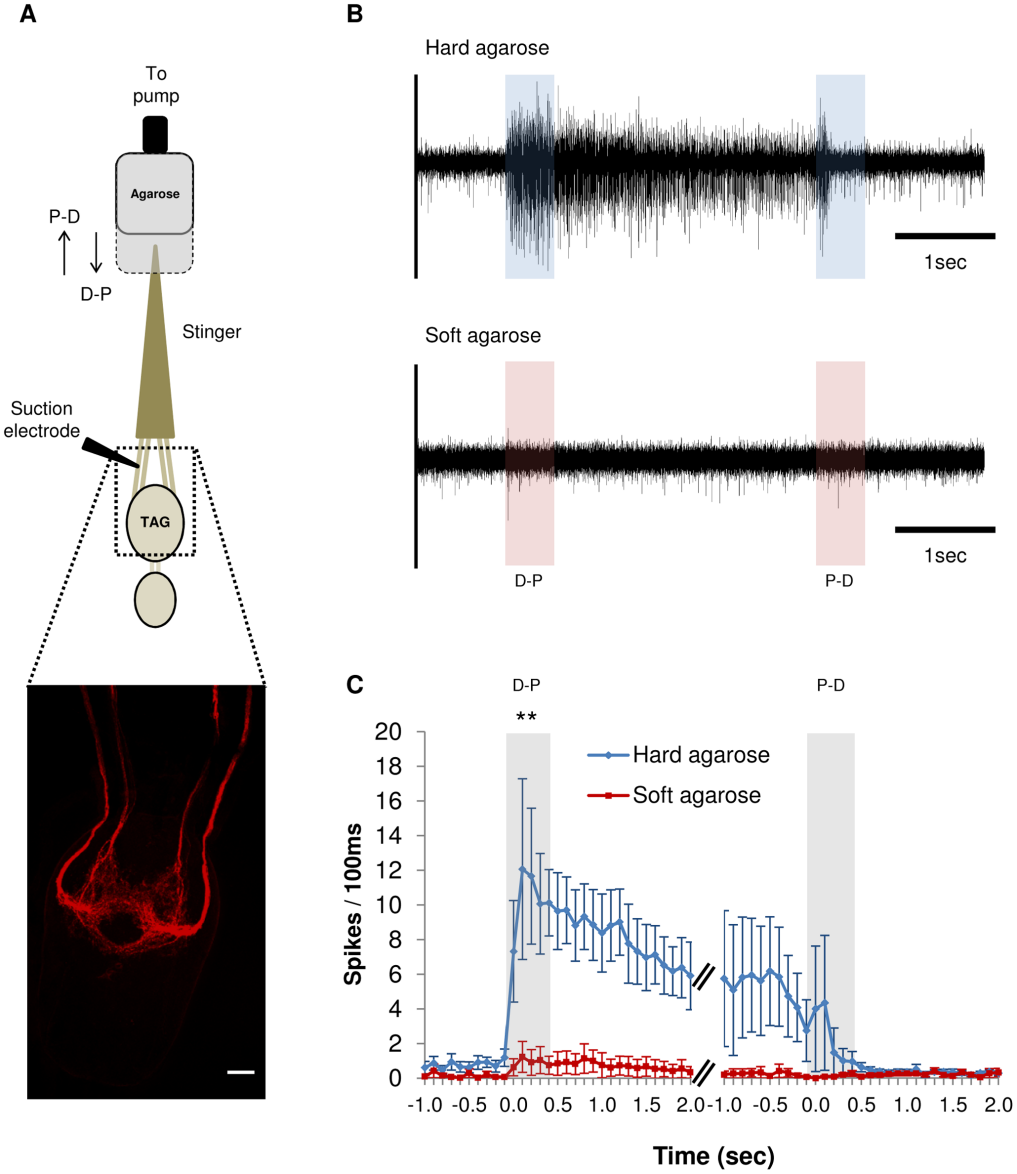
Stinger afferents show spiking activity in response to mechanical stimulation. (**A**) Recording set-up (top view). The wasp’s stinger and terminal abdominal ganglion (TAG) are bathed in saline but with the distal half of the stinger protruding in an approximately 45^0^ angle above the saline. The tip of the stinger is stimulated with either hard agarose or soft agarose in a glass capillary (grey rectangle) which can be moved in the distal-to-proximal (D-P) or in the proximal-to-distal (P-D) direction along the longitudinal axis of the stinger, by means of a peristaltic pump. *En passant* sensory responses are recorded from stinger afferents with a suction electrode placed on the nerves between the stinger and the TAG. A confocal micrograph (bottom) shows Neurobiotin backfills from the tip of the stinger, highlighting sensory afferents ascending from stinger sensilla to the TAG. Scale bar = 50 µm. (**B**) Representative neuronal responses for a stinger isolated from one wasp and stimulated sequentially with hard (2.5%, top) and then with soft (0.5%, bottom) agarose. Left and right shaded areas in each trace represent the duration in which the agarose was actively pushed against (D-P) or pulled away from (P-D) the stinger, respectively. (**C**) Peristimulus time histogram of neuronal activity evoked by hard (blue) or soft (red) agarose stimulation. Data points represent the mean (±SEM) number of sensory spikes within 200 ms time bins. Data is pooled from 5 different wasps, each stimulated at least 10 times in each condition. Left and right grey vertical bars indicate the 500 ms of the stimulus during which the agarose is actively pushed against (D-P) or pulled away from (P-D) the stinger, respectively. **p<0.01 for hard compared with soft agarose during the first 500 ms of the stimulation (t-test; n = 5 wasps).

Because the dorsal valve appears to penetrate the cockroach’s brain, and since CS are distributed on the dorsal but not on the ventral valves, our working hypothesis was that CS sensilla provide at least part of the sensory input required for the complete execution of the head-sting. This requires that singer afferents respond to pressure exerted on the stinger in a manner that complies with the natural stinging behavior. Extracellular electrophysiological recordings from isolated stingers (Fig. 3A) show that, compatible with the identification of CS on the dorsal valve [27] and with the natural stinging behavior (Fig. 1), sensory afferents ascending from the wasp’s stinger to its ventral nerve cord (Fig. 3A, bottom) respond to mechanical stimulation in a direction- and density-dependent manner. First, the firing rate of afferent neurons significantly increases when an agarose pellet is pushed in a distal-to-proximal direction against the tip of the stinger, but to a significantly lesser extent when the agarose is pulled away from the stinger in the opposite direction (Fig. 3B); Second, the firing rate of sensory afferents is significantly higher when a denser agarose (i.e., 2.5% agarose) is pushed against the stinger compared with softer (0.6%) agarose (Fig. 3B). Hence, sensory organs on the stinger may differentiate between different head-borne tissues, based at least on their mechanical properties. Stinger afferents showed no distinct electrophysiological responses to cockroach brain or muscle homogenates, or to 100 mM KCl (n = 6 wasps with at least 3 trials for each condition; data not shown), suggesting that chemoreceptive sensilla are not involved in identifying the brain.

To examine the role of mechanical cues we first ablated stinger cuticular sense organs and their neuronal innervations by dipping the distal end of the stinger in liquid nitrogen. When presented with a cockroach, such ‘stinger-deafferented’ wasps approach and try to sting the cockroach normally; however the duration of the sting dramatically increases from 1.2±0.3 min in untreated wasps to 19.9±10.6 min in deafferented wasps (F = 21.77, p<0.001, n = 7 wasps in each group; data not shown). Next, to directly assess the role of mechanosensory cues in identifying the cockroach’s brain, we performed different surgical procedures in the head of cockroaches prior to a stinging by untreated wasps and quantified the stinging duration. These procedures included (a) removing the brain from the cockroach’s head capsule (similar to experiments presented in [8], [9]); (b) replacing the cockroach’s brain with agarose pellets of different concentrations; (c) injecting tetrodotoxin (TTX) inside the cockroach’s brain; and (d) homogenizing the cockroach’s brain inside the containing head capsule. The thoracic ganglia of all these pre-treated cockroaches were not manipulated; hence, we used the duration of the thoracic sting as an internal control and an indicator of specificity, expecting the experimental manipulations of the cockroach’s brain to not affect the thoracic sting duration.

**Figure 4:**
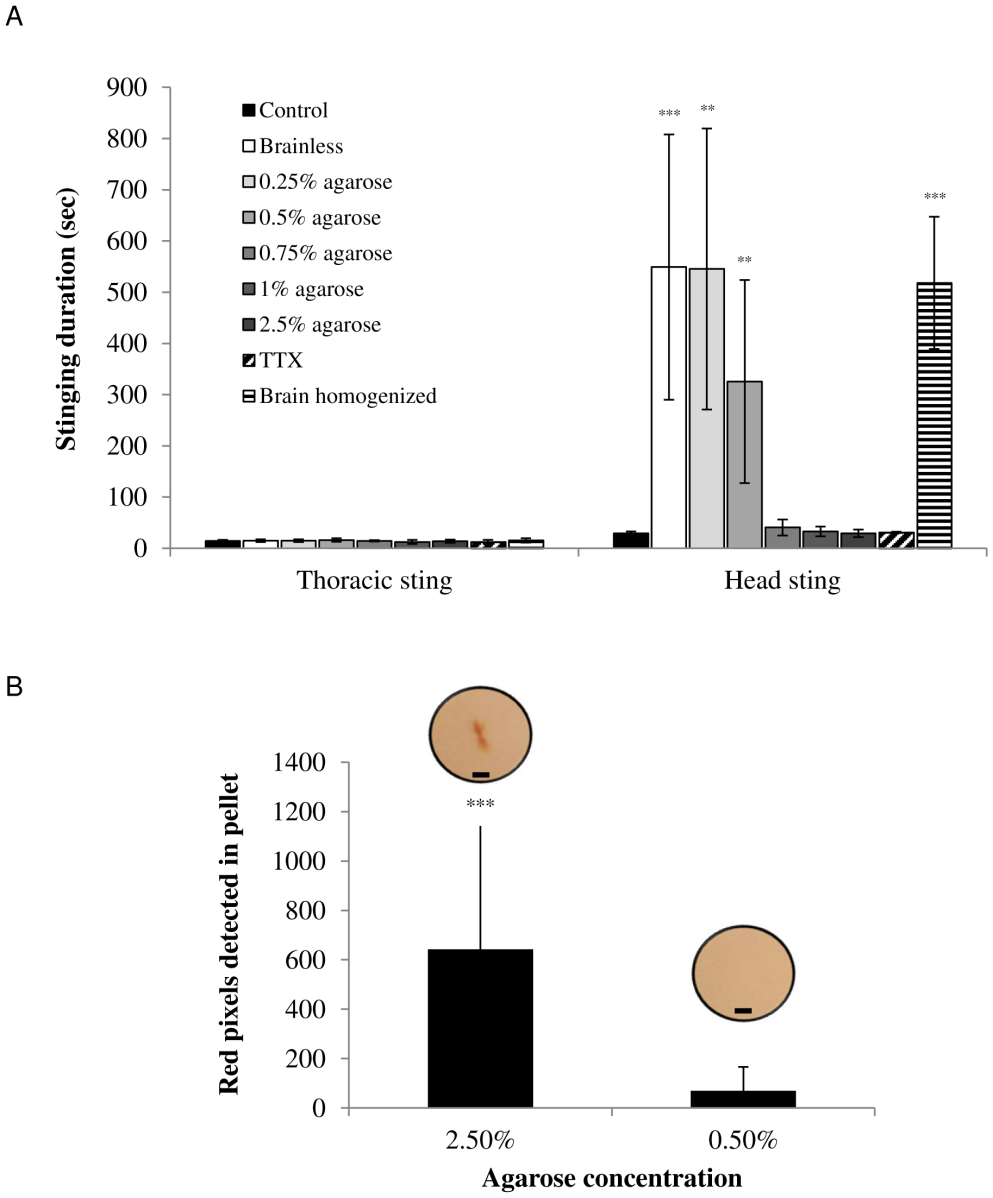
The wasp uses mechanosensory inputs to identify the cockroach’s brain. (**A**) Mean (±SD) stinging duration after different surgical manipulations on the cockroach’s brain prior to a wasp’s sting (see text for details). Control (n = 30); ‘Brainless’ (n = 19); Brain replaced with agarose pellets: 0.25% (n = 12), 0.5% (n = 9), 0.75% (n = 8), 1% (n = 6), 2.5% (n = 12); brain injected with TTX (n = 6); Brain homogenized (n = 5). **p<0.01, ***p<0.001 (Kruskal-Wallis One-Way ANOVA on Ranks versus the control group). (**B**) Number of red pixels, indicative of amount of injected venom in hard (2.5%, n = 10) and soft (0.5%, n = 9) agarose pellets following a wasp’s sting. Inserts are representative photomicrographs of one hard (left) and one soft (right) agarose pellet extracted from cockroach heads immediately after the sting (Scale bars = 0.1 mm). ***p<0.001 (t-test).

Wasps introduced with surgically pre-treated cockroaches usually inflicted the two consecutive stings, first paralyzing the legs with a thoracic sting and then stinging into the head (Fig. 4). However, a Kruskal-Wallis one way ANOVA reveals that whereas the type of pre-treatment does not affect the duration of the thoracic-sting (H = 7.414, p = 0.493), it significantly affects the duration of the head-sting (H = 75.140, p<0.001). Concomitant with the electrophysiological data, the behavioral change depends on the mechanical cues that the stinger encounters inside the cockroach’s head capsule. More specifically, when wasps are introduced with ‘brainless’ cockroaches, from which the brain was completely removed prior to the sting, the head sting is dramatically prolonged often 10-fold and more (Fig. 4A). A similar prolongation of the head-sting occurs when wasps sting cockroaches in which the brain was surgically replaced with a low-density (0.25%–0.75%) agarose pellet. In contrast, the head-sting duration is normal if the cockroach’s brain is replaced with a high-density (0.75%–2.5%) agarose pellet (Fig. 4). The stinging duration appears to reflect events associated with the injection of venom inside the head of the cockroach, as pellets prepared with high-density but not with low-density agarose show traces of venom that can be detected after the sting (Fig. 4B). These results indicate that mechanosensory cues inside the head of the cockroach are sufficient to induce a normal stinging process, and that this sensory input is probably mediated by, at least, mechanosensitive sensilla distributed along the stinger. Furthermore, the head-sting duration is normal for TTX-injected cockroaches but is significantly increased for brain-homogenized cockroaches (Fig. 4A), suggesting that (a) electrical activity in the cockroach’s brain is not necessary for the head-stinging behavior; and (b) mechanical (but probably not chemical) cues are necessary for brain recognition and venom injection. The fact that TTX-injected and brain-homogenized cockroaches were behaviorally indistinguishable and showed the same behavior as “brainless” cockroaches (see [28]) indicates that behavioral cues also do not mediate the brain-recognition process. The specific chemosensory role of DS sensilla is therefore a subject for future study and may include, for example, monitoring venom concentrations proximal to the tip of the stinger during venom injection; determining host health-related factors or hyperparasitism, and more.

## Conclusion

We show that the jewel wasp *Ampulex compressa* uses sensory input from its stinger to differentiate between the brain and other tissues inside the head capsule of its cockroach prey. To identify the brain, the wasp uses (at least) mechanical cues conveyed by sensilla on the stinger, similar to the mechanism other parasitoid wasps use to locate a hidden prey within a surrounding substrate. The ability of the stinger to recognize neuronal tissue inside the head capsule of the cockroach is an exquisite indication of the sensory adaptations that parasitoid hymenopterans have undergone during the ‘evolutionary arms race’ with their hosts [10]–[22], [27].

## Materials and Methods

### Animals

Jewel wasps (*Ampulex compressa* Fabr.) and their cockroach hosts (*Periplaneta americana*) were reared under laboratory conditions as described previously [29]. Wasps used for experiments were 2–6 weeks post eclosion, and all had successfully stung cockroaches at least twice prior to the experiments. Cockroaches used for experiments were adult males reared in crowded conditions.

### Morphology and Ultrastructure

#### Light microscopy of the cockroach head

To observe the different tissues within the cockroach’s head capsule (Fig. 1B) a cockroach brain was fixed overnight in formol-alcohol (100 ml 70% ethanol, 5 ml 40% formaldehyde, 5 ml glacial acetic acid), embedded in agar and sectioned at 0.2 mm with a Leica VT1000S vibratome. Sections at the plane most relevant to the stinging trajectory (see illustration in Fig. 1B) were mounted on slides and observed at 4× magnification under brightfield illumination.

#### Stinger backfills

The tip of the stinger was cut approximately 300 µm from its distal end and the cut end was immersed in de-ionized water for 10 minutes and then in 5% Neurobiotin (in de-ionized water, Vector, Burlingame, CA) for 48 hours at 4°C. The stingers with their associated nerves and abdominal portion of the nerve cords were dissected out and fixed (4% formaldehyde, 3 hours), dehydrated in an ethanol series and washed in propylene oxide (Sigma) for 15 minutes. They were then rehydrated and rinsed 10 minutes in Millonig’s buffer (pH 7.3), incubated at 37°C for 1 hour in a solution of 0.1% collagenase/dispase (Sigma) in Millonig’s buffer, washed in Millonig’s buffer (30 minutes) and in Millonig’s buffer containing 5% normal goat serum and 1% Triton X-100 (15 minutes), and incubated overnight in Millonig’s buffer containing 5% normal goat serum, 1% Triton X-100 and 2 µg/ml avidin-Cy3 (Molecular probes). They were then dehydrated in an ascending ethanol series, cleared in methyl-salicylate and mounted with permount (Fisher Sci. Huston, TX) on a concave microscope slide. The preparations were scanned with a confocal microscope (Zeiss Axiovert200 with LSM). Presented here are representative projections of multiple Z-planes scanning with 10X and 20X objectives.

#### Scanning Electron Microscopy (SEM)

Newborn adult wasps were cold-anesthetized and their stingers isolated, cleared in CCl_4_ overnight, boiled 3 times for 1 minute (each time with fresh CCl_4_) and left overnight in absolute ethanol. The stingers were then rinsed with fresh ethanol, transferred to a 2∶1, 1∶1 and then 0∶1 ethanol: Hexamethyldisilazane solution and air-dried. After mounting on stubs, the specimens were sputter-coated with 10-nm gold/platinum and observed under a Jeol JSM-7400F High Resolution SEM. Overall, stingers from 12 different wasps were studied in detail.

#### Transmission Electron Microscopy (TEM)

Wasps were cold-anesthetized and immersed in Karnowsky’s fixative for 1 h at 4°C. The distal portion of the stingers was then cut, left in fresh fixative for another 3 h at 4°C, rinsed and left overnight in cacodylate buffer and then post-fixed in 1% osmium tetroxide at 4°C for 1 h. After rinsing again with cacodylate buffer, specimens were gradually dehydrated in ethanol and then embedded in Epon-Araldite with propylene oxide as a bridging solvent. Thin sections (60–80 nm) were cut with Leica Ultracut UCT ultra microtome, mounted on formvar-carbon coated grids, double stained with uranylacetate and lead citrate and observed under a Jeol 1230 TEM. Semi-thin sections (0.5–1 µm) were cut on several occasions for observations under a light microscope (e.g., Fig. 2B).

#### Silver nitrate staining

The protocol was similar to [26]. Briefly, intact wasps were immersed for 2 h in 1 M silver nitrate and 70% ethanol, dehydrated in 90% and 100% ethanol and then dissected to remove stingers and antennae (which were used as a positive control for tracer penetration into chemosensory sensilla). The specimens were then cleaned overnight in xylene and observed under a light microscope.

### Electrophysiology

A wasp was cold-anesthetized and its abdomen mounted dorsal-side-up on a recording platform. The ventral nerve cord was exposed in cold saline [30] in which a ground electrode was placed. The sheath of the stinger was then removed to expose the cuticular shaft, which protruded outside the saline in an approximately 45^0^ angle relative to the platform. The preparation was continuously perfused with aerated saline at 24°C throughout the recording sessions and, in preliminary experiments, a drop of 0.01% Janus Green B was added to the preparation for 20–30 sec to better visualize the neuronal tissue [31] and the afferent nerves were identified empirically. After carefully exposing the stinger afferents, a suction electrode was used to record extracellular *en passant* spiking neuronal activity while the tip was mechanically stimulated with agarose (Fig. 3A). For stimulation, agarose prepared at different concentrations was filled into a glass capillary connected through an electrode holder (which allowed changing the capillary in different stimulation conditions) to the flat surface of a 3 ml syringe plunger. The syringe itself was filled with water and mounted on a micromanipulator, such that the nozzle of the syringe was connected through silicone tubing to a peristaltic pump (Pump P-1, Pharmacia Biotech). In this setup, activation of the pump in one direction pushed the plunger (and the attached agarose-filled capillary) forward, whereas activation in the other direction pulled the plunger backwards, along the same longitudinal axis. This simple device allowed controllable movements of the capillary along the longitudinal axis of the stinger at a constant velocity (1.5 mm/sec, controlled by the peristaltic pump) and in both directions (i.e., distal-to-proximal (D-P) or proximal-to-distal (P-D)). During stimulation, the capillary was first placed close to the tip of the stinger and then pushed via the peristaltic pump in the D-P direction. This ‘forward’ motion was maintained for 500 ms after the stinger established contact with the agarose, and was then stopped such that the capillary remained stationary for 3–5 additional seconds. Then, the capillary was retracted in the P-D direction along the same axis and at the same velocity, until the stinger exited the agarose. In each recording session the wasp’s stinger was stimulated at least 10 times in each direction at either condition (soft or hard agarose, alternatively). Sensory spikes were amplified with a differential amplifier (DAM80, World Precision Instruments Inc., Sarasota, FL) and acquired, sorted and analyzed (offline) with Spike2 data acquisition system and software (CED, Cambridge, UK). A 100 ms bin size was selected for the peristimulus time histogram (PSTH) and the number of spikes in each bin was averaged for all stimulations in each wasp, and then pooled together for all wasps. A t-test was used to compare the number of spikes occurring in the first 500 ms following stimulation between different stimulation conditions.

We used a slightly different setup to test the response of stinger sensilla to cockroach brains or mandibular muscles homogenized in 100 mM KCl. In this setup, the stinger was dissected as described above and its distal 500 µm were then inserted into silicone tubing through a small hole perforated in the tubing. A peristaltic pump (see above) was used to circularly pass solutions (3 cycles in each wasp) across the tip of the stinger in the following order: KCl, brain homogenate, KCl, muscle homogenate. A bipolar electrode connected to a DAM80 amplifier (see above) was inserted through the tubing and placed near the tip of the stinger to allow extracellular recording of sensory spikes.

### Behavioral Experiments

#### Quantification of stinging durations

A cockroach was introduced into a wasp’s home-cage until the wasp stung the cockroach voluntarily. Thoracic and head-stinging durations were measured with a stopwatch by an experienced observer.

#### Stinger deafferentation

To ablate sensory organs on the stinger and their neuronal innervations, wasps were confined in a milking device [8] and were stimulated to sting a piece of parafilm. As the stinger protruded from the far side of the parafilm, liquid nitrogen was dripped over the distal half of the stinger to ablate all sense organs at this location. After a one-day recovery period, the treated wasps were allowed to freely sting intact cockroaches and the duration of the stinging sequence was measured.

#### Cockroach surgical procedures

Some of the surgical procedures used in this work are detailed in [28]. Briefly, cockroaches were cold-anesthetized, a flap was opened in their dorsal head cuticle to expose the brain and the procedure (see below) was performed. The flap was then closed and sealed with beeswax to prevent hemolymph outflow. All cockroaches were allowed to recover for 2–4 h, at the end of which their behavior was quantified [28] for 15 min before they were introduced to the wasp.

#### Brain removal (‘Brainless’ cockroaches)

The procedure was similar to that described in [28]; after exposing the brain, the circumesophageal, optical and antennal nerves were cut with fine microscissors and the brain was completely removed from the head capsule with fine forceps. Care was taken to minimize damage to non-neuronal tissue inside the head capsule.

#### Agarose pellets

Commercial agarose was prepared at different concentrations (as indicated) in distilled water to which, in some instances, the pH indicator Neutral Red was added (10%, pH adjusted to 8 with NaOH to receive a yellowish color). The melted agarose was then applied onto parafilm as 3 µl droplets using a micropipette, and allowed to cool in this pellet-like form. The cockroach’s head was then opened and the brain removed as described above. The formed cavity was immediately filled with the agarose pellet, and the head was closed as described above. When a pH indicator was used, the pellet was removed from the cockroach’s head capsule immediately after the sting and photographed in a brightfield microscope using a 20× objective. The image was then processed with a custom-made MATLAB color filter, such that red pixels (filter parameters determined empirically) were automatically counted to indicate the presence of the acidic venom inside the pellet.

#### Brain homogenization

Two approaches were alternatively used: (1) the brain was removed from the head capsule as described above, placed in a small drop of cockroach saline, desheathed with fine microscissors and forceps and returned into the head capsule with a micropipette; (2) the brain was homogenized with microscissors inside the head capsule, without removing it from the cockroach. The two methods yielded statistically similar results (data not shown) and were thus combined for simplicity.

#### TTX injections

Tetrodotoxin (0.1 mM in cockroach saline; Sigma-Aldrich) was injected directly into the middle of the brain (100 nL/brain) with a nanovolumetric injector (NVI-570 A/V, Medical Systems, Greenvale, NY). Only cockroaches that were behaviorally indistinguishable from 'brainless' cockroaches (as described in detail in [28]) were used for stinging experiments. Injections of saline into the brain did not affect neither the wasp’s nor the cockroach’s behavior (data not shown).

#### Statistical analysis

We used a One-Way ANOVA for normally distributed data and a Kruskal-Wallis One-Way ANOVA on Ranks for non-normally distributed data (as indicated in the text). Dunn’s posthoc tests for multiple comparisons versus the control group were used to compare stinging durations. A t-test was used to compare number of stained pixels in agarose pellets.
